# Functional molecular expression of nature killer cells correlated to HBsAg clearance in HBeAg-positive chronic hepatitis B patients during PEG-IFN α-2a therapy

**DOI:** 10.3389/fimmu.2022.1067362

**Published:** 2022-11-21

**Authors:** Weihua Cao, Huihui Lu, Luxue Zhang, Shiyu Wang, Wen Deng, Tingting Jiang, Yanjie Lin, Liu Yang, Xiaoyue Bi, Yao Lu, Lu Zhang, Ge Shen, Ruyu Liu, Min Chang, Shuling Wu, Yuanjiao Gao, Hongxiao Hao, Mengjiao Xu, Xiaoxue Chen, Leiping Hu, Yao Xie, Minghui Li

**Affiliations:** ^1^ Department of Hepatology Division 2, Beijing Ditan Hospital, Capital Medical University, Beijing, China; ^2^ Department of Infectious Diseases, Miyun Teaching Hospital, Capital Medical University, Beijing, China; ^3^ Department of Obstetrics and Gynecology, Wuhan Children’s Hospital (Wuhan Maternal and Child Healthcare Hospital), Tongji Medical College, Huazhong University of Science and Technology, Wuhan, China; ^4^ Infectious Disease Department, Xuanwu Hospital, Capital Medical University, Beijing, China; ^5^ Department of Hepatology Division 2, Peking University Ditan Teaching Hospital, Beijing, China

**Keywords:** nature killer cells, chronic hepatitis B, PEG-IFN α-2a, hepatitis B virus, HBsAg loss

## Abstract

**Objective:**

To explore whether the frequencies and functional molecules expression of Natural Killer cells (NK cells) are related to hepatitis B surface antigen (HBsAg) disappearance in hepatitis B e envelope antigen (HBeAg)-positive patients with chronic hepatitis B (CHB) throughout peginterferon alpha-2a (PEG-IFN α-2a) treatment.

**Methods:**

In this prospective research, HBeAg-positive patients with CHB received PEG-IFN α-2a treatment, completing 4-year follow-up. After PEG-IFN α-2a treatment, undetectable HBV DNA, HBsAg loss, and HBeAg disappearance were defined as functional cure. Proportions of NK, CD56^dim^, CD56^bright^, NKp46^+^, NKp46^dim^, NKp46^high^, and interferon alpha receptor 2 (IFNAR2)^+^ NK cells, and the mean fluorescence intensity (MFI) of NK cell surface receptors IFNAR2 and NKp46 were detected.

**Results:**

66 patients were enrolled into the study in which 17 patients obtained functional cure. At baseline, hepatitis B virus desoxyribose nucleic acid (HBV DNA) titer in patients with functional cure was remarkably lower than that in Non-functional cure group. Compared with baseline, HBV DNA levels, HBsAg levels, and HBeAg levels significantly declined at week 12 and 24 of therapy in patients with functional cure. At baseline, the negative correlation between CD56^bright^ NK% and HBV DNA and the negative correlation between CD56^dim^ NK% and HBV DNA was showed; CD56^bright^ NK% and IFNAR2 MFI in patients with functional cure were remarkably higher than those in patients without functional cure. After therapy, CD56^bright^ NK% and NKp46^high^ NK% in patients with functional cure were higher than those in patients without functional cure. In Functional cure group, after 24 weeks of treatment NK%, CD56^bright^ NK%, IFNAR2 MFI weakly increased, and NKp46^high^ NK% and NKp46 MFI significantly increased, meanwhile, CD56^dim^ NK% and NKp46^dim^ NK% decreased. Only NKp46 MFI increased after therapy in patients without functional cure.

**Conclusion:**

The lower HBV DNA load and the higher CD56^bright^ NK% before therapy, and the higher the post-treatment CD56^bright^ NK%, IFNAR2 MFI, NKp46^high^ NK%, the easier to achieve functional cure.

## Introduction

Hepatits B virus (HBV) infection is still a public health problem worldwide; especially chronic HBV infection which is very harmful to human health (1, 2). Therefore, eliminating hepatitis B has become the topical issue over recent years. Annually, the rate of spontaneous HBsAg clearance is approximately 1% in natural history of chronic HBV infection (3, 4). It is urgent to explore the optimal treatment strategies for CHB. At present, the most effective treatment to delay the progress of CHB is antiviral therapy. However, if we want to enhance living quality and lengthen the survival period to the most extent, “functional cure”, identified as undetectable HBV DNA, persistent HBsAg loss, whether or not accompanied by HBsAg serological conversion (5, 6), is pursued as the treatment end point (7, 8). Currently, by NAs therapy, CHB patients achieve HBsAg loss at a lower rate <1% per year, while HBsAg clearance rate is the highest in CHB patients with HBeAg positivity, who receive IFN therapy (9–12). Therefore, improving the hosts’ immune response through PEG-IFN optimal treatment can more effectively eliminate the virus and even achieve clinical cure (13–15).

As a crucial component in innate immunity, NK cells have a key part in eliminating viruses as well as immunologic defence (16). NK cells consist of CD56^bright^ NK cells and CD56^dim^ NK cells. Generally, CD56^bright^ NK cells have a key role mainly in triggering the specific immune response by secreting immunomodulation cytokines and CD56^dim^ NK cells have a key role in directly eliminating target cells (17, 18). The function of NK cells is induced by an array of inhibiting or active surface receptors (19). NKp46, as the activated receptor on the surface of NK cells, is a key receptor to activate the killing activity of NK cells. IFNAR2 expressed on the surface of NK cells plays an antiviral role when the expression was upregulated and can make IFN-α more easily to activate NK cells, helping to eliminate viruses. IFNAR2 deficiency results in dysregulation of NK cell functions (20). In humans, some studies showed that NK cells were activated in patients with acute HBV infection (21), with the increased expression of activation markers including NKp46, the increased CD56^bright^ NK cells subpopulation, the cytotoxicity and IFN-γ production of NK cells (22–24). However, the activities of NK cells are significantly damaged in CHB patients. HBsAg and HBeAg directly inhibit function of NK cells by multi-signal pathways correlated to the activation of NK cells (25–27). The up-regulation of inhibition receptors and the down-regulation of activation receptors, as the phenotypic characteristics of NK cells, are commonly found in chronic infection, leading to the impairment of effector functions, which may lead to the persistence of viruses (28).

PEG-IFN α increases the inhibing effect of NK cells on regulatory T cells by IFN-γ in CHB (29). After IFN treatment, remarkable increase in expansion and activation of CD56^bright^ NK cells, IFN-γ production, and augmentation of NK cell activating receptors (NKp46 and NKp30) expression are observed (30, 31). After PEG-IFN treatment, the recovery of CD56^bright^ NK cells function and expansion of percentages of NKp46^+^ and CD56^bright^ NK cells were revealed in inactive HBsAg carriers and CHB patients with NAs therapy (32, 33). The percentage and functional molecular expression of NK cells correlated to HBsAg clearance in treatment-naive HBeAg-positive CHB patients during PEG-IFN α-2a therapy are relatively rare. Thereby, the aim of the research is to probe in treatment-naive CHB patients with HBeAg positivity, whether proportion of NK cells and functional molecules expression on the surface of NK cells are correlated to functional cure throughout PEG-IFN α-2a treatment.

## Materials and methods

### Subjects

In the nonrandomized, single center, prospective cohort study, 66 HBeAg (+) CHB patients with naïve therapy were enrolled from October 2014 to November 2017 visiting the Liver Disease Center of Beijing Ditan Hospital, Capital Medical University. The inclusion criteria were detectable HBV DNA, HBsAg positive for > 24 weeks, HBeAg positive, and continuously abnormal alanine aminotransferase (ALT) for > 12 weeks, or liver puncture indicating apparent inflammation. Exclusion criteria were the followings (1): co-infected with other viruses (HDV, HIV, or HCV et al) (2); HCV, HIV or syphilis antibodies positive (3); complicated with other liver diseases consisting of fatty liver, hepatic cirrhosis, hepatocellular carcinoma, autoimmune hepatitis, metabolic liver disease, alcoholic liver disease et al (4); use of hormone or immunomodulatory medication. All patients signed written informed consent. Our study was approved by Beijing Ditan Hospital Institutional Review Board and registered at clinicaltrials.gov (ID: NCT03208998).

All patients received individualized treatment and followed up for 4 years. As follows, patients were treated with PEG-IFN α-2a alone, 180 μg subcutaneous injection weekly for 24 weeks. After 24 weeks of treatment, HBV DNA negative patients would continue PEG-IFN α-2a single-drug treatment. However, patients who didn’t achieve HBV DNA negative needed PEG-IFN α-2a combined with tenofovir or entecavir treatment, and specific need for treatment with entecavir or tenofovir depended on previous medical history and health status of those patients. In the follow-up therapy, on the basis of the decrease of HBsAg and HBeAg levels, if HBsAg and HBeAg levels continued to decrease, or even HBsAg clearance, patients were continuely treated with PEG-IFN α-2a consolidation therapy for 12 weeks-24 weeks, then drug withdrawal. Nevertheless, if HBsAg and HBeAg levels didn’t decrease continuously and remained at the platform period, PEG-IFN α-2a needed to be stopped, then the maintenance monotherapy of ETV or TDF would be continued. HBsAg/HBsAb levels, HBeAg/HBeAb levels, HBV DNA titers and ALT were tested before therapy and per 12 weeks after treatment till HBsAg disappeared. Measurements of the frequency and functional molecule expression of NK cells were performed before therapy and at 12 weeks and 24 weeks of therapy.

Functional cure was as follow: persistent HBsAg disappearance, whether or not accompanied by HBsAg serological conversion, HBeAg loss with or without seroconversion, and HBV DNA negative. Conversely, it was defined as non functional cure.

### Assessment of clinical parameters

Serum HBsAg/HBsAb and HBeAg/HBeAb levels were quantitated by Abbott Architect i2000 Detection Reagent (Abbott Diagnostics, Abbott Park, IL); HBV DNA titers were detected by Roche Cobas AmpliPrep/Cobas TaqMan 96 full-automatic real-time fluorescent quantitative PCR detection reagent (Roche, Pleasanton, CA); and biochemical indexes were measured by Hitachi 7600 full-automatic biochemical analyzer (Hitachi, Japan). The detection range of HBsAg was 0.05-250 IU/ml, HBeAg ≥ 1 S/CO was positive, and HBeAb<1S/CO was positive. The lower limit of serum HBV DNA detection value was 20 IU/ml. The upper limit of normal ALT value was 40U/L.

### Detection of NK cells

Peripheral venous blood of all patients was collected by EDTA anticoagulant violet tube before therapy (baseline), at the 12 and 24 weeks after PEG-IFN therapy. Cell phenotyping stained with four monoclonal Antibodies (mAbs) including Mouse anti-Human-CD3-PerCP (Becton-Dickinson, USA; clone: SP34-2), Mouse anti-Human CD56-APC (Becton-Dickinson, USA clone: B159), anti-Human CD335 (NKp46)-PE (Biolegend, USA; clone: 9E2), and Anti-Human IFNAR2-FITC (Sino Biological, Beijing, China; clone: 07), were analyzed by flow cytometry (FACS Caliburflow Cytometer, USA) within 4 hours after collection, as shown in **Supplementary Figure 1**. The proportions of NK, CD56^dim^ and CD56^bright^, NKp46^+^, and IFNAR2^+^ NK cells, and MFI of NKp46 and IFNAR2 were analyzed by FlowJo 7.6.1 software. Expression of surface receptors NKp46 and IFNAR2 on NK cells represented NK cell function.

### Statistical methods

In the research, statistical data were processed by SPSS 26 (SPSS Inc., Chicago, USA). continuous variables were represented by mean ± standard deviation or median (Q1, Q3). Repeated-measures analysis of variance was used for changes of the indexes at three observation time points, and p values for multiplicity were ajusted by Bonferroni, and p < 0.016 was considered as statistical significance. Independent sample t-test was used to analyze normal distribution data and Mann-Whitney nonparametric U test was used to analyze skewness distribution data between two groups. Spearman’s correlation was applied for the association of variables. We used binary logistic regression analysis to analyze independent factors of functional cure. P < 0.05 was considered to be statistically significant.

## Results

### Baseline features of various indicators in patients with CHB

In our research, 66 HBeAg (+) patients with CHB received PEG-IFN-α-2a individually, following up for 4 years, lincluding 40 males and 26 females. Patients with undetectable HBV DNA and HBsAg disappearance were considered as functional cure, on the contrary, as non functional cure, with 17 cases obtaining functional cure. Among them, 3 patients with HBsAg clearance lost the detection of the frequency and functional molecules expression of NK cells at 12 weeks or 24 weeks of therapy, and 9 patients without HBsAg clearance lost the detection of the frequency and functional molecules expression of NK cells at 12 weeks or 24 weeks of therapy.

The baseline serological virological indicators of the two groups were compared. We found that the serum HBsAg, HBeAg and ALT had no remarkable statistical differences, respectively, between two groups (**Table 1**), but the HBV DNA titers were lower in functional cure group than those in non functional cure group (*P* = 0.001) in **Table 1**. As shown in **Table 1**, at 12 weeks and 24 weeks of therapy, HBV DNA (*P* = 0.034(12w), *P* = 0.005(24w)), HBsAg (*P* = 0.000(12w), *P* = 0.000(24w)), as well as HBeAg (*P* = 0.030(12w), *P* = 0.011(24w)) in patients without functional cure were remarkebly higher than those in patients with functional cure. Before therapy, CD56^bright^ NK/NK% (*P*=0.000), CD56^dim^ NK/NK% (*P*=0.000) and IFNAR2 MFI (*P*=0.043) had significant statistical differences between functional cure group and non functional cure group (**Table 2**).

**Table 1:** Comparison of clinical characteristics.

TItem	All patients N = 66	Baseline		*P* value	12w		*P* value	24w		*P* value
		Non-functional cure group N = 49	Functional cure group N = 17		Non-functional cure group N = 40	Functional cure group N = 14		Non-functional cure group N = 40	Functional cure group N = 14	
Male/Female	40/26	32/17	8/9		25/15	7/7		25/15	7/7	
Age(yrs)	30 (20–51)	30 (20–51)	32 (24–49)	0.476	30 (20–51)	31 (24–49)	0.976	30 (20–51)	31 (24–49)	0.976
HBsAg (log10 IU/mL)	3.864 ± 0.678	3.911± 0.667	3.727± 0.710	0.833	3.377 ± 0.702	1.720± 1.470	0.000	3.196 ± 0.802	1.509± 1.683	0.000
HBV DNA (log10 IU/mL)	7.070 ± 1.136	7.329 ± 0.908	6.324 ± 1.405	0.001	4.095± 1.555	3.146 ± 1.560	0.034	2.084± 0.760	1.233 ± 0.879	0.005
HBeAg (PEIU/mL)	879.460 (372.835, 1396.338)	878.350 (369.29,1350.645)	880.570 (441.715, 1409.685)	0.809	59.895 (16.438,406.53)	21.895 (0.353, 88.013)	0.030	18.920 (5.728, 82.250)	1.855 (0.325, 17.475)	0.011
ALT level(U/L)	253.100 (129.525, 355.550)	234.700 (127.400, 357.400)	273.000 (135.250, 371.150)	0.337	67.150 (49.450, 82.050)	64.250 (20.50,101.825)	0.608	44.250 (28.150,56.425)	28.700 (22.350,67.975)	0.424

ALT, alanine aminotransferase; HBsAg, hepatitis B surface antigen; HBeAg, hepatitis B e antigen. P value: Non-functional cure group vs Functional cure group. P value <0.05, statistical significance.

**Table 2:** and surface receptors NKp46 and INFAR2 expression of NK cells at baseline between functional cure group and non-functional cure group.

Item	All subjects (n = 66)	Non-functional cure group (n = 49)	Functional cure group (n = 17)	*P* value^*^
Gender(M/F)	40/26	32/17	8/9	
NK(%)	11.024± 6.011	11.361± 6.201	10.054 ± 5.486	0.491
CD56^bright^ NK/NK(%)	14.367 ± 7.661	12.466 ± 6.739	19.848 ± 7.695	0.000
CD56^dim^ NK/NK(%)	85.633 ± 7.661	87.534 ± 6.739	80.152 ± 7.695	0.000
IFNAR2^+^ NK/NK(%)	4.998 ± 2.788	5.115 ± 2.809	6.664 ± 2.782	0.450
IFNAR2 MFI	21.750 (15.175, 27.200)	19.900 (14.450, 25.900)	26.300 (18.375, 39.800)	0.043
NKp46^+^ NK/NK(%)	90.765 ± 6.621	90.363 ± 7.509	91.924 ± 2.646	0.687
NKp46^bright^ NK/NK(%)	17.886 ± 8.506	17.342 ± 8.911	19.456 ± 7.224	0.194
NKp46^dim^ NK/NK(%)	82.114 ± 8.506	82.658 ± 8.911	80.544 ± 7.224	0.194
NKp46 MFI	91.600 (63.775, 138.750)	88.400 (57.200, 134.500)	107.000 (87.050, 159.500)	0.086

^*^Non-functional-cure group vs. Functional cure group. In all analyses, P value < 0.05, statistical significance.

We used Binary logistic regression analysis to analyze baseline clinical indicators, proportions and and functional molecules expression of NK cells in two groups, but no independent influencing factors of functional cure were observed.

### Correlation of the parameters of NK cells with virological indicators and ALT at baseline

**Figure 1:**
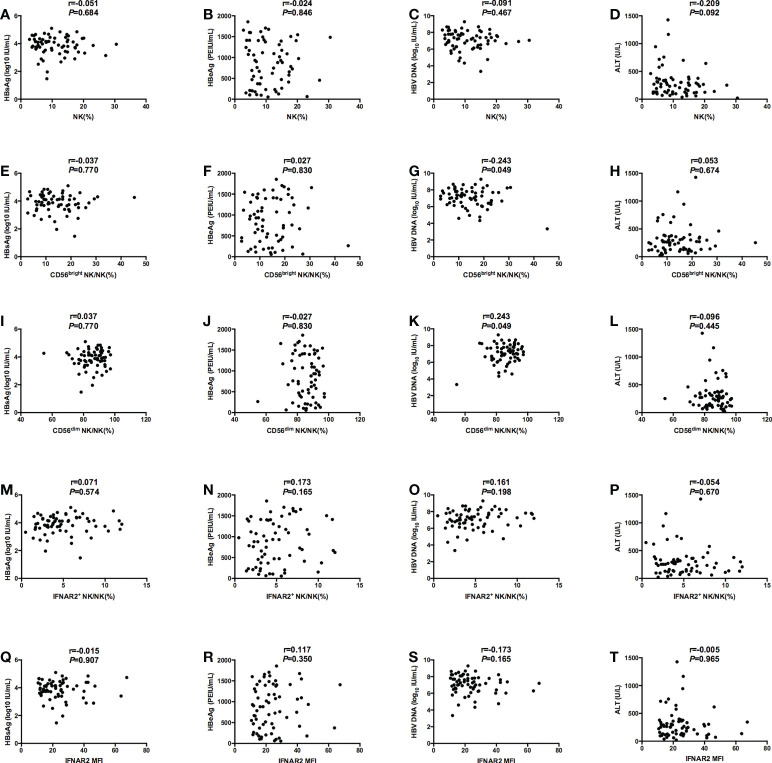
Correlations between NK cell frequency or function (NK%, CD56bright NK/NK%, CD56dim NK/NK%, IFNAR2+ NK/NK%, and IFNAR2 MFI) and serological and virological indicators (HBsAg, HBeAg, and HBV DNA, ALT). **(A-D)** Correlations between NK% and serological and virological indicators (HBsAg **(A)**, HBeAg **(B)**, and HBV DNA **(C)**, ALT **(D)**). **(E-H)** Correlations between CD56bright NK/NK% and serological and virological indicators (HBsAg **(E)**, HBeAg **(F)**, and HBV DNA **(G)**, ALT **(H)**). **(I-L)** Correlations between CD56dim NK/NK% and serological and virological indicators (HBsAg **(I)**, HBeAg **(J)**, and HBV DNA **(K)**, ALT **(L)**). **(M-P)** Correlations between IFNAR2+ NK/NK% and serological and virological indicators (HBsAg **(M)**, HBeAg **(N)**, and HBV DNA **(O)**, ALT **(P)**). **(Q-T)** Correlations between IFNAR2 MFI and serological and virological indicators (HBsAg **(Q)**, HBeAg **(R)**, and HBV DNA **(S)**, ALT **(T)**). P < 0.05 was considered to be statistically significant.

At baseline, we performed the correlation analysis on the frequency of NK cells, the expression of surface functional molecules NKp46, INFAR2 and HBV virological characteristics. We found that there were no correlations between NK cell frequency or function (NK%, IFNAR2+ NK/NK%, and IFNAR2 MFI) and serological and virological indicators (HBsAg, HBeAg, and HBV DNA, ALT). (**Figures 1A-F, H-J, L-T**) And there were no correlations between the expression of surface functional molecules NKp46 and virological indicators.. The negative correlation between CD56^bright^ NK% and HBV DNA (r=-0.243, *P* = 0.049) (**Figure 1G**) and the negative correlation between CD56^dim^ NK% and HBV DNA (r=0.243, *P* = 0.049) (**Figure 1K**) was observed.

### NK cells function and CHB function cure

**Figure 2:**
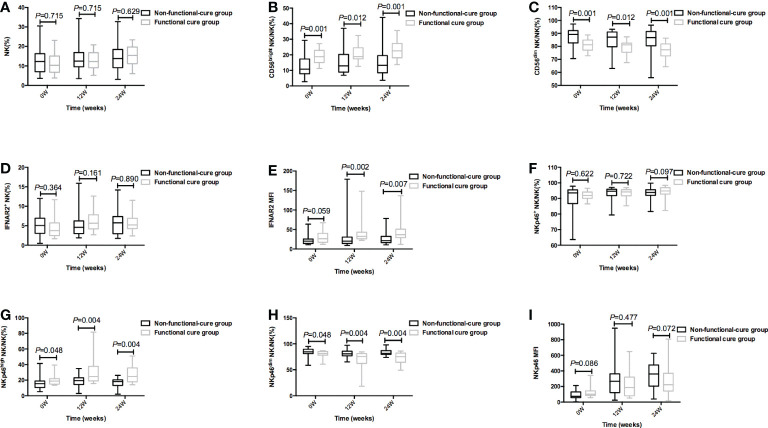
During PEG-IFNα-2a therapy, the frequency of NK cells and surface functional molecules [NK% **(A)**, CD56bright NK/NK% **(B)**, CD56dim NK/NK% **(C)**, IFNAR2+ NK/NK% **(D)**, IFNAR2 MFI **(E)**, NKp46 NK/NK% **(F)**, NKp46high NK/NK% **(G)**, NKp46dim NK/NK% **(H)**, and NKp46MFI **(I)**] between Non-functional-cure group and Functional cure group were compared before therapy and at 12 weeks and 24 weeks of therapy, respectively. P<0.05 was statistically significant.

The baseline P values in **Table 2** and **Figure 2** were different, because **Table 2** showed the total number of patients in the functional cure group and the non functional cure group, and at 12 and 24 weeks of treatment, 3 patients in the functional cure group and 9 patients in the non functional cure group were lost to follow-up. **Figure 2** showed the statistical analysis after excluding the lost patients. At baseline and at 12 weeks and 24 weeks of therapy, CD56^bright^ NK/NK% (*P* = 0.001;(0w) *P* = 0.012 (12w); *P* = 0.001(24w)), NKp46^high^ NK/NK% (*P* = 0.048(0w); *P* = 0.004(12w); *P* = 0.004 (24w)) in functional cure group were markedly higher than those in non functional cure group. CD56^dim^ NK/NK% (*P* = 0.001(0w); *P* = 0.012(12w); *P* = 0.0001 (24w)), NKp46^dim^ NK/NK% (*P* = 0.048(0w); *P* = 0.004(12w); *P* = 0.004(24w)) in functional cure group were markbly lower than those in non functional cure group in **Figure 2**.

**Figure 3:**
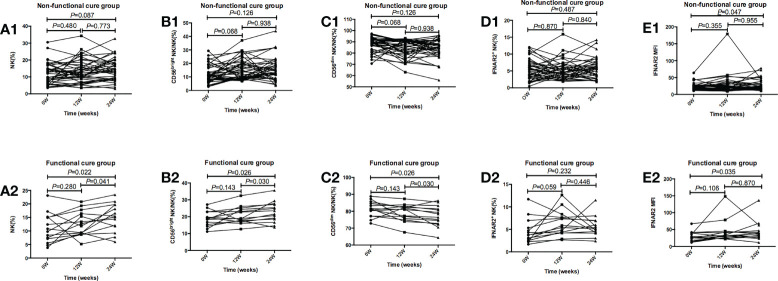
Tendencies of NK cells frequency and function, including NK%, CD56bright NK/NK%, CD56dim NK/NK%, IFNAR2+ NK/NK%, and IFNAR2 MFI at baseline and after 12 and 24 weeks of treatment in the Functional cure group and Non-functional-cure group. **(A1-E1)** Tendencies of NK% **(A1)**, CD56bright NK/NK% **(B1)**, CD56dim NK/NK% **(C1)**, IFNAR2+ NK/NK% **(D1)**, and IFNAR2 MFI **(E1)** at baseline and after 12 and 24 weeks of treatment in the Non-functional-cure group. **(A2-E2)** Tendencies of NK%(A2), CD56bright NK/NK% **(B2)**, CD56dim NK/NK% **(C2)**, IFNAR2+ NK/NK% **(D2)**, and IFNAR2 MFI **(E2)** at baseline and after 12 and 24 weeks of treatment in the Functional cure group. p values for multiplicity were ajusted by Bonferroni, and P < 0.016 was dentifified as signifificant statistical difference.

In **Figure 3**, in functional cure group, our results showed NK%, CD56^bright^ NK/NK% and IFNAR2 MFI weakly increased after 24 weeks of treatment, CD56^dim^ NK/NK% decreased after treatment. However, *p* values for multiplicity were ajusted by Bonferroni, so *p*>0.016 has no significant statistical significance.

**Figure 4:**
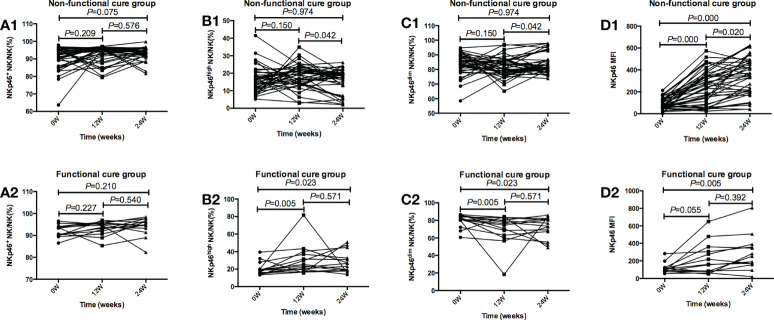
Tendencies of NK cells frequency and function, including NKp46 NK/NK%, NKp46high NK/NK%, NKp46dim NK/NK%, and NKp46MFI at baseline and after 12 and 24 weeks of treatment in the Functional cure group and Non-functional-cure group. **(A1-D1)** Tendencies of NKp46 NK/NK% **(A1)**, NKp46high NK/NK%(B1), NKp46dim NK/NK% **(C1)**, and NKp46MFI **(D1)** at baseline and after 12 and 24 weeks of treatment in the Non-functional-cure group. **(A2-D2)** Tendencies of NKp46 NK/NK% **(A2)**, NKp46high NK/NK% **(B2)**, NKp46dim NK/NK% **(C2)**, and NKp46MFI **(D2)** at baseline and after 12 and 24 weeks of treatment in the Functional cure group. p values for multiplicity were ajusted by Bonferroni, and P < 0.016 was dentifified as signifificant statistical difference.

As shown in **Figure 4**, in functional cure group, NKp46^high^ NK/NK% after 12 weeks of treatment was dramatically higher than that at baseline (*P*=0.005), NKp46^dim^ NK/NK% after 12 weeks of treatment was markedly lower than that before therapy (*P*=0.005), and NKp46 MFI after 24 weeks of treatment (*P*=0.005) was dramatically higher than that before treatment. In non functional cure group, NKp46 MFI was significantly higher after treatment (*P*=0.000) than that before treatment.

## Discussion

NK cells, as the main components of innate immunity, play a crucial part in viral elimination, meanwhile, they participate in the process of liver tissue damage (34). CD56^bright^ and CD56^dim^ NK cells are the subsets of NK cells. The main function of CD56^bright^ NK cells is to secrete cytokines, including TNF-α, IFN-γ and others, and play an antiviral role through non cytotoxic effects. CD56^dim^ NK cells kill viral infected liver cells through cytotoxic effects mediated by granzyme and perforin (35). NKp46 is an activated receptor on NK cell surface. In acute hepatitis B infection, expression of activiting receptor NKp46 is up-regulated, which resulted in enhancement of NK cell activity (23); The surface of NK cells also expresses the receptor IFNAR2, and the up regulation of IFNAR2 expression can make IFN-a more easier to activate NK cells, play an antiviral role, and help to eliminate viruses. However, several studies have reported that in chronic HBV infection, dyfunction of NK cells is revealed, which is not conducive to the elimination of HBV (36). Therefore, we need to choose the optimal treatment to restore and even enhance the immune cell function, so as to enhance the hosts’ immune response to the viruses, which may lead to HBsAg loss and achieve functional cure. In a variety of treatment methods for CHB, PEG-IFN has antiviral role and immunomodulatory effect. Previous studies have shown that PEG-IFN therapy can enhance function of NK cells and facilitate virus clearance (13, 31–33). NK cells were the important part of innate immunity. Interferon triggered innate immunity, which took effect at 12 weeks of treatment and peaks at 24 weeks. The early to mid-term efficacy of interferon on natural killer cell induction determined the subsequent efficacy, so the changes in the charateristics of NK cells at 12 and 24 weeks of treatment were tested (25). In our research, we explored in CHB patients with HBeAg positivity, whether proportions and function of NK cells were related to functional cure throughout IFN treatment.

Our results demenstrated that HBV DNA titers in patients without HBsAg clearance were significantly higher than those in patients with HBsAg clearance, meanwhile, HBV DNA, HBsAg, and HBeAg dramatically declined during IFN therapy in patients with functional cure, which correspond to previous studies demonstrating the lower HBV DNA, the lower transcription and replication activities of HBV DNA, which leads to the decrease of viral protein production and even the reduction of cccDNA (37), the easier to achieve HBsAg loss and even functional cure.

Previous studies suggested that HBsAg and HBeAg could inhibit the function of NK cells resulting in insufficient immune response without the virus clearance (25–27). Negative correlation between HBsAg level and percentage of CD56^bright^ NK cells was found in previous study (31). Even though we didn’t observe the correlation between CD56^bright^ NK cells and HBsAg, our result demenstrated that the baseline CD56^bright^ NK was negatively correlated with HBV DNA. Tjwa ET et al. found that the reduction of viral load improved the function of NK cells in chronic HBV infected patients (38). Some studies suggested that the decrease of viral load achieved through antiviral treatment partially reshaped phenotype and function of NK cells (13, 17), which indirectly indicated that HBV DNA had an inhibiting role on NK cells. In our study, we found that HBV DNA before therapy in patients with functional cure was dramatically lower than that in patients without non functional cure, as well as CD56^bright^ NK increased after PEG-IFN therapy. Therefore, our results were consistent with the above conclusions. Our study suggested that the baseline CD56^bright^ NK% at baseline and post-treatment in the functional cure group were higher than those in the non-functional cure group, meanwhile, CD56^bright^ NK% increased, therefore, the higher baseline CD56^bright^ NK%, the more likely to obtain functional cure. After PEG-IFN treatment, the immunity was up-regulated, and the immune response in the functional cure group was better,which made CD56^bright^ NK increase. Thus, patients with high baseline level and good immune response after treatment were more likely to achieve functional cure. Our data also showed that CD56^bright^ NK was negatively correlated with HBV DNA at baseline and significantly higher in patients with functional cure than those without functional cure, meanwhile it also increased in the former goup after PEG-IFN therapy, which were in line with previous studies indicating that the ability of CD56^bright^ NK cells to secrete IFN-γ was significantly enhanced during HBeAg seroconversion, which proved the importance of IFN-γ secreted by CD56^bright^ NK cells in controlling virus replication (39). Not only did IFN-α have direct antiviral role, but also made the up-regulation of TRAIL expression on ths surface of NK cells, activated CD56^bright^ NK cells, and increased IFN-γ secretion (40). Activated CD56^bright^ NK cells may be conducive to lead to HBsAg decrease or HBsAg disappearance and even obtain functional cure.

Receptors NKp46 and IFNAR2 on NK cell surface play crucial effects in chronic HBV infection. Type I interferon receptor (IFNAR) has been involved in the progression of CHB. IFNAR2 plays a vital part in initiation of type I interferon-induced JAK-STAT signaling and activation of STATs (41). IFNAR2 deficiency results in dysregulation of the function of NK cells (20). Our data showed that after PEG-IFN treatment, the expression of reporters IFNAR2 on the surface of NK cells in patients with functional cure significantly increased, which suggested that the up-regulation of IFNAR2 made NK cells more easily activated by IFN-α and contributed to clearing HBV. NKp46, as the activating receptor of NK cells, may regulate the activity of NK cells in the suppression of HBV replication and HBV-related liver injury, meanwhile, it is crucial for the activation of NK cells in chronic HBV infection (42). Our resluts showed that after therapy, the frequency of NKp46^high^ NK cells and surface reporters IFNAR2 and NKp46 expression on NK cells in patients with HBsAg loss significantly increased, which was consist with some studies suggesting that in patient with CHB, the expression of IFNAR2 and NKp46 on the surface of NK cells elevated after PEG-IFN therapy (32, 33), NKp46^high^ NK cells may be closely correlated to HBV clearance, and up-regulated expression of NKp46^high^ is beneficial to the clearance of HBV.

In conclusion, this study adds to our knowledge of the relevance of functional cure and frequency and function of NK cells in CHB patients with PEG-IFN α-2a treatment. Our results demonstrate the lower HBV DNA before therapy, the higher CD56^bright^ NK% at baseline, and the higher CD56^bright^ NK%, IFNAR2 MFI, NKp46^high^ NK% after therapy, the easier to achieve HBsAg clearance. Functional cure is dependent on low HBV DNA titer, the recovery and enhancment of NK cells function at the early phase of PEG-IFN α-2a therapy. These conclusions may be conducive to idnetify those easier to obtain functional cure and optimizing therapeutic strategies for CHB to pursue functional cure.

## Data availability statement

The original contributions presented in the study are included in the article/**Supplementary Material**. Further inquiries can be directed to the corresponding authors.

## Ethics statement

The studies involving human participants were reviewed and approved by the Ethics Review Committee of Beijing Ditan Hospital of Capital Medical University. The patients/participants provided their written informed consent to participate in this study.

## Author contributions

WC, YX, and ML made contribution to conception and design of the study. HL, LuxZ, SWang, WD, TJ, YLin, LY, GS, XB, YLu, LuZ, RL, MC, SWu, YG, MX, HH, LH, and XC organized the data. WC, YX, and ML guided statistical analysis. WC wrote the first manuscript. HL, LuxZ, SWang, WD, YX, and ML edited and modified the English manuscript. ML submitted the modified version. All authors contributed to the article and approved the submitted version.

## Funding

The study was supported by Beijing Hospitals Authority Clinical medicine Development of special funding support (XMLX 202127); The capital health research and development of special (2022–1–2172); National Science and Technology Major Project of China (2017ZX10201201-001-006, 2017ZX10201201-002-006, 2018ZX10715-005-003-005); High-level Public Health Technical Personnel Training Program of Beijing Municipal Health Commission (2022–3–050); The Digestive Medical Coordinated Development Center of Beijing Hospitals Authority (XXZ0302 and XXT28); Project supported by Beijing science and technology commission (Z211100002921059).

## Conflict of interest

The authors declare that the research was conducted in the absence of any commercial or financial relationships that could be construed as a potential conflict of interest.

The reviewer SR declared a shared parent affiliation with the authors to the handling editor at the time of review.

## Publisher’s note

All claims expressed in this article are solely those of the authors and do not necessarily represent those of their affiliated organizations, or those of the publisher, the editors and the reviewers. Any product that may be evaluated in this article, or claim that may be made by its manufacturer, is not guaranteed or endorsed by the publisher.

## Supplementary material

Supplementary Figure 1 Using FlowJo software for CD3-CD56+NK cell image analysis to illustrative dot plots related to the FACS data.
