# Supplementary figures and images for: Functional molecular expression of nature killer cells correlated to HBsAg clearance in HBeAg-positive chronic hepatitis B patients during PEG-IFN α-2a therapy
Source: Front Immunol. 2022 Nov 21;13:1067362. doi: 10.3389/fimmu.2022.1067362 (PMC9720173; doi:10.3389/fimmu.2022.1067362)

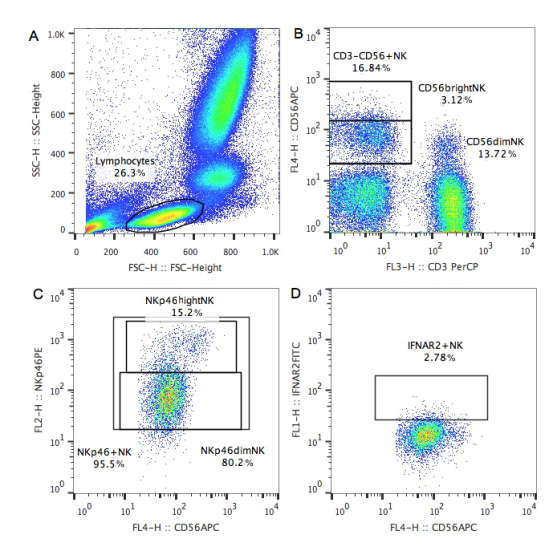

Supplement: Supplementary Figure 1 — Using FlowJo software for CD3-CD56+NK cell image analysis to illustrative dot plots related to the FACS data. [file Image_1.tif]
